# Effect of Recycled Asphalt Mixture and Solid Waste-Based Solidification Materials on Performance of Cold-Regenerated Asphalt Mixture

**DOI:** 10.3390/ma17205099

**Published:** 2024-10-18

**Authors:** Benan Shu, Guodong Zeng, Yunlong Ma, Yanfei Ren, Maocong Zhu

**Affiliations:** 1 National Engineering Research Center of Highway Maintenance Technology, Changsha University of Science & Technology, Changsha 410114, China; 2 Foshan Transportation Science and Technology Co., Ltd., Foshan 528000, China; zls2698@163.com (G.Z.); 234505@whut.edu.cn (Y.R.); zhumaocong1@163.com (M.Z.); 3 Fojiaoke Tiannuo (Guangdong) Materials Co., Ltd., Foshan 528000, China

**Keywords:** recycled asphalt mixture, solidification materials, solid waste, regeneration, material properties, property

## Abstract

In this study, an aging asphalt mixture was regenerated by a waste-based rejuvenator and cemented by solid waste-based solidification materials (SSMs). A splitting test, wheel tracking test, and three-point bending test were conducted to evaluate the properties of the regenerated asphalt mixture (RAM). The results reveal that the properties of the asphalt mixture were not diminished or were moderately enhanced by the 30% substitution of RAP. With the substitution of RAP to 100%, the splitting tensile strength, dynamic stability, and splitting strength ratio were decreased by 13%, 15%, and 5%, respectively. With the 100% substitution of SSMs for cement, the compressive strength, dynamic stability, flexural strain, and splitting strength ratios of the RAM were increased by 40%, 32%%, 14%, and 8%, respectively. The lightweight components can be supplemented, and low-temperature deformation and interlayer flowability can be improved by the incorporation of the rejuvenator. The generation of hydrated calcium silicate and ettringite for SSMs is greater than those of cement. The massive generation of ettringite has been observed to increase the solid phase volume by 120%, which may facilitate a more complete filling of the remaining pores in the RAM due to water evaporation. The regeneration and cement on green and the high performance of the rejuvenator and the SSM markedly enhanced RAM performance.

## 1. Introduction

Asphalt pavements are widely used in road construction due to their favorable properties, including flexibility, durability, and cost-effectiveness [1,2]. However, these materials are susceptible to degradation over time due to environmental factors such as ultraviolet radiation, temperature fluctuations, and traffic loads. This aging process results in the hardening, reduced ductility, and increased brittleness of the asphalt binder, which can subsequently result in cracking, raveling, and other forms of distress [3,4,5,6]. As a result, maintaining the structural integrity and functionality of aging asphalt pavements has become a significant challenge for civil engineers and transportation agencies.

With the increasing focus on sustainability and environmental conservation, traditional methods of pavement rehabilitation, such as complete removal and replacement, are being scrutinized for their resource intensity and environmental impact. In response, cold recycling has emerged as a promising alternative. This method not only reduces the need for new materials but also helps in conserving energy and minimizing carbon emissions associated with asphalt production. However, the performance of cold recycling asphalt mixtures is heavily influenced by the properties of the aged asphalt binder and the effectiveness of the additives utilized in the recycling process [7,8,9,10].

To address this issue, rejuvenators have been developed and employed in the regeneration of aging asphalt binders. Rejuvenators work by replenishing the lost volatiles and restoring the plasticity and ductility of the binder, making it more suitable for modern traffic conditions and environmental stresses. Recent studies have highlighted the effectiveness of various types of rejuvenators, including bio-based oils, petroleum-based products, and synthetic polymers [11,12,13,14,15]. Iwama et al. [16] studied the effect of two types of oil-based rejuvenators on the durability of RAP mixtures compared with that of a hot mixed asphalt (HMA) mixture. The results reveal that the RAP mixtures did not demonstrate a decline in retained strength, whereas the HMA mixtures exhibited a substantial reduction in strength with conditioning. Moreover, the RAP mixtures demonstrated a superior residual service life compared to the HMA mixtures post-conditioning. Schwettmann et al. [17] found that the impact of different types of rejuvenators on RAP and artificially aged asphalt samples varies. As a general rule, artificially aged asphalt samples display less evidence of aging than RAP. Furthermore, the aging behavior of pure regenerants can also influence the regeneration and utilization of RAP.

In addition to rejuvenators, the incorporation of cement as an additive in cold-recycled asphalt mixtures has gained attention in recent years. Various studies have shown that the inclusion of cement can lead to improved properties [18,19,20,21,22]. Ji et al. [23] found that the content of cement can be adjusted in order to enhance the early strength of cold-recycled asphalt mixtures. A cement-to-water ratio can be employed as the primary controlling factor for the early strength of cold-recycled emulsified asphalt mixtures. It can be posited that the optimal range for the proportion of cement to water is between 0.6 and 1.0. Furthermore, the cement content should be maintained between 1.2 and 1.5%, while the moisture content should be controlled between 3.8 and 4.3%. Dong et al. [24] investigated the incorporation of low cement content into cold-recycled mixtures, which was observed to exert minimal influence on their strength. It may be posited that the composition and strength of these mixtures are predominantly influenced by factors other than the cement content alone. At the microscopic level, the interface transition zone displays characteristics that indicate interweaving of cement hydration products with asphalt mortar, resulting in the formation of a network structure. In comparison to the adhesion observed at the aggregate–asphalt interface, the bond between cement hydration products and aggregates is notably weaker.

While cement can enhance the performance of the cold-recycled asphalt mixture, the production of cement itself is a highly polluting and carbon-intensive industry. Consequently, it would be prudent to reduce or eliminate its use. The objective of this article is to develop cleaner regenerants and cementitious materials with the aim of improving the performance of RAP and achieving full recycling of RAP. In view of this, this article presents the development of a waste-based rejuvenator (comprising primarily waste cooking oil, waste rubber powder, and modified graphene oxide) for the restoration of aged asphalt performance, as well as the creation of a solid waste-based solidification material (primarily consisting of steel slag, fly ash, and an activator) to supplant cement in the improvement of regenerated asphalt mixture performance. A systematic study was conducted to investigate the effects of RAP and SSMs on the properties of regenerated asphalt mixtures, including mechanical properties, high-temperature properties, low-temperature properties, and water stability. This research presents a novel methodology for the environmentally sustainable and high-performance regeneration of aged asphalt mixtures.

## 2. Materials and Methods

### 2.1. Materials

A solid waste solidification material (SSM) was investigated and developed by Shu et al. [25,26,27]. This SSM is a kind of hydraulic cementing material, which is mainly composed of industrial solid waste, steel slag, and fly ash in the proportion of 95%, and an alkaline activator accounted for 5%. The basic properties of the SSM are shown in Table 1, and this SSM meets the requirements of the standard “Soft Soil Solidifying Agent” (CJ/T526-2018, Beijing, China, 2018).

The rejuvenator was self-developed as follows: The WCO (procured from a restaurant in Foshan City) and waste rubber powder ((WR) purchased from Linshou Shifeng Mining Processing Factory) were subjected to precise weighing, yielding a mass ratio of 5:5. The WR was subsequently partitioned into five equal-mass fractions. At the outset, the WCO was heated to a temperature of 250 °C. This was followed by the addition of the initial batch of WR. Following the aforementioned addition, a five-minute stirring period was initiated at a speed of 350 rpm. Subsequently, the second batch of WR was added to the aforementioned mixture, and the same stirring speed and time were maintained. Subsequently, the entirety of the WR contents were incorporated into the aforementioned mixture, which was agitated for one hour at 350 rpm. Subsequently, the modified graphene oxide (modified by KH-550) with the contents of 0.1% was introduced gradually and mixed for an additional five minutes at 350 rpm, thereby resulting in the creation of the rejuvenator.

The aged asphalt mixture was extracted from a principal thoroughfare in Foshan, Guangdong. The road has been in use for a period of 5 years, during which time it has developed significant cracks and ruts. The moisture content of the aged asphalt mixture was 0.5%. Given that the test regenerant is primarily based on the content of the aged asphalt, the asphalt aggregate ratio of the old asphalt mixture was determined to be 4.2% through the use of the Abson asphalt recovery method. The utilization of the old asphalt mixture does not entail the application of heating. In the event that the unextracted aggregate size is excessive, the aggregate size resulting from the extraction process is calculated in accordance with the specifications outlined in the mix design. In this study, a medium-grained gradation was employed. In the event that the gradation of the milling material failed to meet the requisite specifications, limestone gravel aggregate was introduced. Subsequently, the gradation was determined based on the results of the raw material screening and the engineering design grading range, following adjustments and optimizations through the addition of new aggregates, and the RAM gradation is shown in Table 2. It can be discerned that there was a notable enhancement in the proportion of recycled aggregate particles in each gradation measuring below 9.75 mm. The slow-cracking and slow-setting cationic emulsified asphalt is in accordance with the specified requirements “Technical specification for cold in plant recycling by emulsified asphalt in asphalt pavement” (DB 37/T 3566-2019, Jinan, China, 2019). The optimal moisture content and the optimal asphalt dosage are slightly adjusted with the RAP content. The proportion of rejuvenator to the mass of aged asphalt is 10%, and the proportion of cement to the mass of the aged asphalt mixture is 2.0%.

### 2.2. Methods

**Figure 1:**
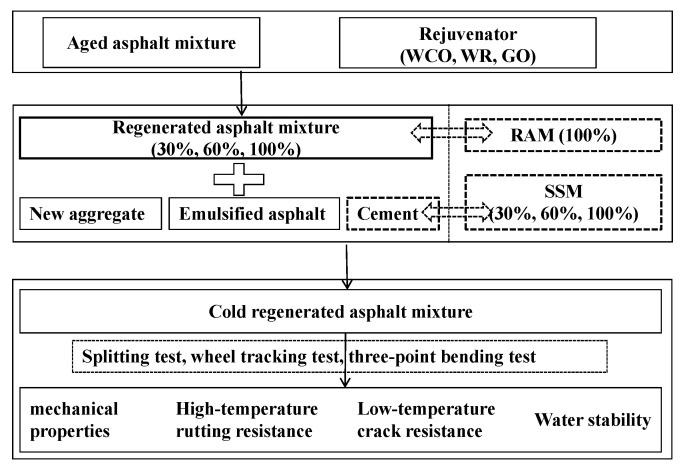
Workflow diagram of this study.

Figure 1 shows the workflow diagram of this study.

**Figure 2:**
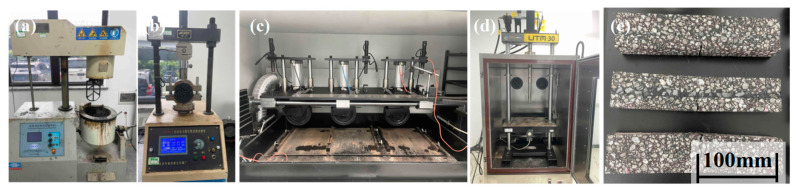
Experimental devices of (**a**) mixing, (**b**) water stability, (**c**) wheel tracking test, and (**d**) three-point bending test and (**e**) asphalt mixture beams for low-temperature testing.

Figure 2 shows the experimental devices for mixing and testing recycled asphalt mixtures. A splitting test, wheel tracking test, and three-point bending test were conducted in accordance with the specification “Standard Test Methods of Bitumen and Bituminous Mixtures for Highway Engineering” (JTG E20-2011, Beijing, China, 2011).

An indirect tensile strength evaluation of the recycled asphalt mixture was conducted using a splitting test. The standard Marshall samples of φ = 101.6 mm × h = 63.5 mm were prepared. The samples were placed in an incubator set at a temperature of 15 °C for a duration of six hours, after which the splitting test was conducted using a universal testing machine. The temperature of the apparatus was maintained at 15 °C throughout the test, and the loading rate was set at a rate of 50 mm/min.

A uniaxial compression test was employed to assess the compressive strength of the cold-recycled asphalt mixture. The samples were placed in an environmental box maintained at 20 °C for a period of six hours prior to undergoing a uniaxial compression test conducted using a universal testing machine. The temperature of the testing apparatus was maintained at 20 °C, and the load was applied at a rate of 2 mm/min.

For the wheel tracking test, a standard rut plate specimen measuring 300 mm × 300 mm × 50 mm was fabricated. At the outset of the test, the specimen was situated within a temperature-controlled environment box maintained at 20 °C for a period of six hours prior to commencing the rut test. During the test, the grinding wheel exerted pressure of 0.7 MPa on the specimen while the test environment temperature was set at 60 °C.

For the low-temperature bending test, 250 mm × 35 mm × 30 mm trabecular samples were prepared by cutting a prepared rut plate into the aforementioned dimensions and placed in a thermostatic freezing solution at a temperature of −10°C for a period of four hours. Following this, bending test was carried out utilizing a UTM-30 (IPC, Sydney, Australia) microcomputer-controlled electronic universal testing machine. The test temperature was set to −10 °C, and the loading rate was 2 mm/min.

For the water stability test, the standard Marshall samples were initially prepared and subsequently divided into two groups. One group was subjected to a dry splitting test following the preservation of the samples at a constant temperature of 15 °C for a period of 6 h. The other group was subjected to wet splitting test firstly following the submergence of the samples in water at 25 °C for 23 h, followed by a 1 h immersion in water at 15 °C. The wet splitting strength was then determined. The test temperature was maintained at 15 °C, with a loading rate of 50 mm/min. Three sets of test blocks were tested in parallel under the same experimental conditions.

## 3. Results and Discussion

### 3.1. Cleavage and Compressive Strength

**Figure 3:**
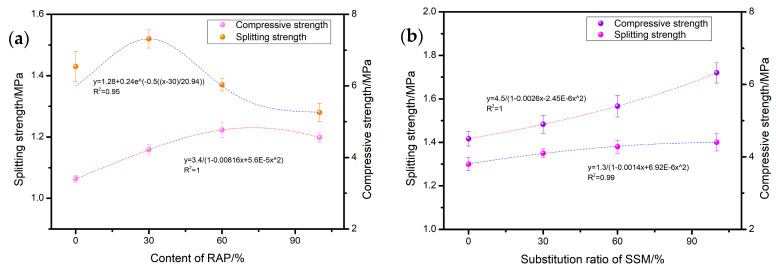
Effect of (**a**) RAP and (**b**) SSMs on mechanical properties.

Figure 3 illustrates the impact of RAP and SSMs on the mechanical properties of the mixture. From Figure 3a, it can be observed that as the RAP content is augmented, the splitting tensile strength of the mixture initially displays a modest increase. At a 30% RAP content, the splitting tensile strength exhibited a 7% augmentation to 1.52 MPa, after which it demonstrated a gradual reduction in strength. As the proportion of RAP in the mixture increased to 60%, the compression strength of the mixture exhibited an increase of 37% to 4.8 MPa. However, subsequent to this point, a decline in compression strength was observed. The replacement of 100% RAP resulted in a decrease of approximately 13% in the splitting performance. The findings of the present study align with those of previous research, indicating that the incorporation of high RAP content into asphalt mixtures leads to a reduction in splitting strength and an enhancement in compressive strength [28].

As illustrated in Figure 3b, the compressive performance of the asphalt mixture exhibits a marked enhancement with the incremental substitution of SSM for cement content. Upon substitution of 100% SSM for cement, a 40% increase in compressive strength of the mixture is observed, reaching 6.5 MPa. An increase in the proportion of SSM in the mixture is accompanied by a marginal enhancement in its splitting strength. The alteration in mechanical properties may be attributed to the restricted scope of RAP substitution. As a consequence of the swelling and degradation of rubber particles within the rejuvenator, the rejuvenator displays superior bonding characteristics in comparison to the matrix asphalt. Consequently, the restricted incorporation of recycled asphalt pavement (RAP) can enhance the mechanical characteristics of asphalt mixtures to a certain degree. As the RAP content increases, the refinement of aggregates within the RAP results in changes to the mixture’s gradation, which subsequently weakens the interlocking effect between aggregates and, therefore, the mixture’s splitting strength. The enhancement in compactness serves to augment the compressive strength of the mixture. The replacement of cement with SSMs can result in an additional enhancement of the compressive strength of the mixture. This is due to the fact that SSMs generate a greater quantity of hydrates, such as ettringite, in comparison to cement hydration. This is advantageous as it allows for a more effective filling of the pores that are left in the mixture as a result of water evaporation. The addition of SSMs to the mixture results in a marked improvement in its compactness, accompanied by a substantial rise in compressive strength.

### 3.2. High-Temperature Rutting Resistance

**Figure 4:**
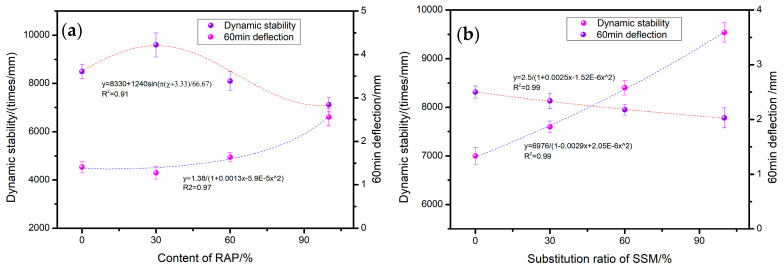
Effect of (**a**) RAP and (**b**) SSMs on high-temperature rutting resistance.

The impact of RAP and SSMs on the high-temperature rutting resistance of the mixture is illustrated in the accompanying Figure 4. As illustrated in Figure 4a, the dynamic stability of the mixture initially increases and subsequently declines with increasing RAP content. The greatest increase is observed at a dosage of 30% (9600 times/mm), with an average enhancement of 13% observed across all tested dosage levels. Upon replacement with 100% RAP, the dynamic stability of the asphalt mixture exhibited a 15% reduction, reaching 7200 times/mm in comparison to the original asphalt mixture. Additionally, a 65% increase in deformation was observed after 60 min. This data pattern is in accordance with the findings of the research conducted by Huang et al. [29].

The incorporation of SSMs into the composition of a fully recycled asphalt mixture has been demonstrated to result in an incremental enhancement in the mixture’s dynamic stability, contingent upon the extent of SSM replacement as shown in Figure 4b. Additionally, a corresponding reduction in the deformation amount has been observed over the course of 60 min. The replacement of cement with SSM 100% results in a 32% increase, up to 9500 times/mm, in the dynamic stability of the asphalt mixture and a 20% reduction, down to 2 mm, in deformation after a period of 60 min. The rationale behind the modification in high-temperature anti-rutting capability can be attributed to the incorporation of a waste rubber powder component within the rejuvenator, which effectively augments the asphalt’s high-temperature performance. The superior high-temperature performance of recycled asphalt, when a small quantity of RAP is incorporated, serves to significantly enhance the anti-rut characteristics of the resulting mixture. As the proportion of RAP in the mixture increases, the softening effect of the light component in the rejuvenator and deterioration of the interlocking structure of the mixture due to the refining of recycled aggregates become the predominant factors, resulting in increased deformation and a decline in anti-rutting performance. Due to its superior cementitious effect in comparison to cement, in addition to its capacity to more effectively fill pores and enhance compactness, the overall stiffness of the mixture is enhanced, which consequently leads to an improvement in the mixture’s rutting resistance.

### 3.3. Low-Temperature Crack Resistance

**Figure 5:**
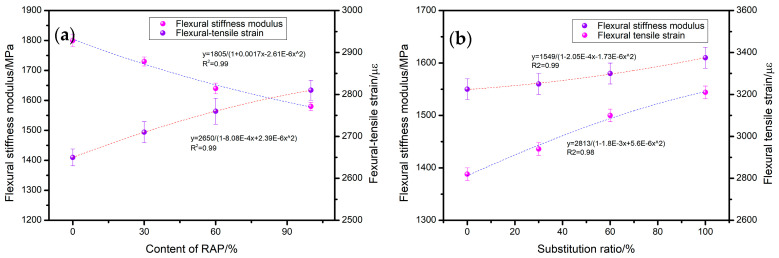
Effect of (**a**) RAP and (**b**) SSMs on low-temperature crack resistance.

The impact of RAP and SSM on the low-temperature cracking resistance of recycled asphalt mixtures is illustrated in the accompanying Figure 5. As illustrated in Figure 5a, an increase in RAP dosage is accompanied by a gradual decline in the flexural modulus of the mixture. Upon replacement of the RAP by 100%, a decrease in the flexural modulus of the mixture by 14% to 1550 MPa was observed. The flexural strain of the mixture demonstrated a gradual increase with the replacement of RAP, reaching a 6% increase at the 100% RAP replacement level. The primary reason for this is that the waste edible oil present in the rejuvenator is capable of supplementing the light components of the aged asphalt, thereby enhancing the low-temperature performance of the mixture. Additionally, the rubber components of the rejuvenator exhibit excellent elasticity and deformation ability in low-temperature conditions, facilitating the release of stress and the prevention of microcrack development. As the quantity of SSM substitution is increased, the bending stiffness modulus and bending tensile strain of the mixture demonstrate a range of increases (as shown in Figure 5b). Upon replacement of 100% of the cement with SSMs, the aforementioned values demonstrate increases of 5% and 14%, respectively. The use of SSMs in construction results in a denser overall structure of the mixture due to its superior hydration and cementing properties and pore-filling effects compared to cement. The mixture is capable of resisting greater bending and tensile stresses, which in turn results in increased bending stiffness modulus and bending and tensile strain of the mixture.

### 3.4. Water Stability

**Figure 6:**
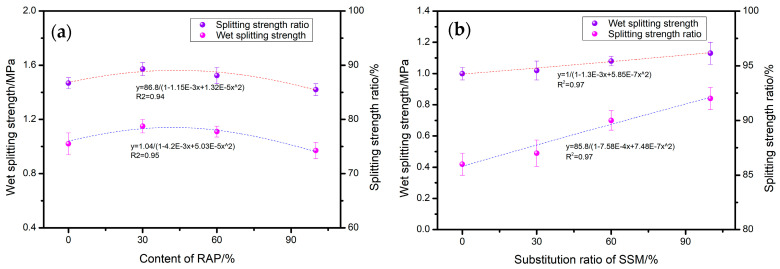
Effect of (**a**) RAP and (**b**) SSMs on water stability.

The impact of RAP and SSMs on the water stability of recycled asphalt mixtures is illustrated in Figure 6. As illustrated in Figure 6a, an increase in RAP content initially results in a slight enhancement in both the wet splitting strength and the splitting strength ratio of the mixture. However, this is subsequently followed by a slight decline. At a RAP substitution ratio of 30%, the values increase to 1.15 MPa and 88%, respectively. Upon reaching 100% RAP substitution, both values decrease to 0.95 MPa and 85%. The findings of the present study align with those of previous research, indicating that the incorporation of high RAP content into asphalt mixtures can lead to a reduction in water stability [30].

The primary reason for this is the excellent water stability (with the addition of 30% RAP, the splitting strength ratio was increased by 11%) inherent to the developed rejuvenator, which, in conjunction with modified graphene, facilitates an interlayer flow of asphalt, thereby enabling the regenerated asphalt to establish optimal contact and adhesion with the aggregate. This effect serves to mitigate the adverse consequences of the pre-existing aged asphalt that is attached to the surface of recycled aggregates. As illustrated in Figure 6b, the incorporation of SSMs in lieu of cement results in enhanced water stability of the mixture. Upon replacement of cement by SSMs in quantities reaching 100%, the wet splitting strength and splitting strength ratio of the mixture were observed to be 1.1 MPa and 92%, respectively, representing increases of 10% and 8%, respectively. The primary reason for this is that SSMs are better able to fill the internal pores of the mixture than cement, thereby reducing the invasion path and space of moisture. This results in an improvement in the water stability performance of the mixture.

### 3.5. Regeneration Mechanism Analysis

**Figure 7:**
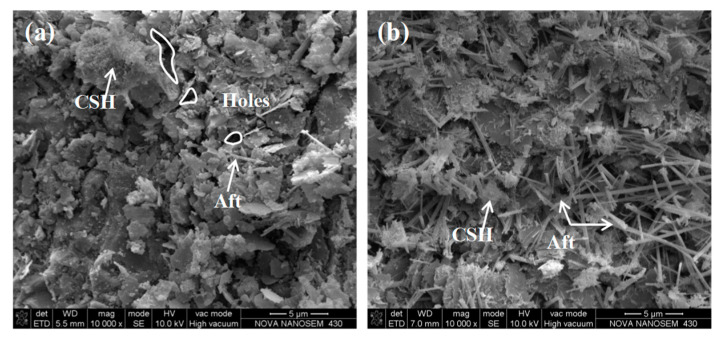
Micro morphology of (**a**) cement and (**b**) SSM after hydration reaction.

The microstructure resulting from the SSM hydration reaction is illustrated in Figure 7. The principal hydration products of cement and SSMs are hydrated calcium silicate and needle-shaped ettringite. The primary distinction between the two is the presence of a reduced number of cement hydrates and a lack of complete bonding and coverage of the solid particles. A minor quantity of ettringite was discerned, accompanied by a considerable number of unfilled pores. In contrast, the SSM hydration system exhibited a substantial coverage of gelatinous calcium silicate gel, with a considerable quantity of needle-shaped ettringite filling the pores, resulting in a highly dense overall structure.

**Figure 8:**
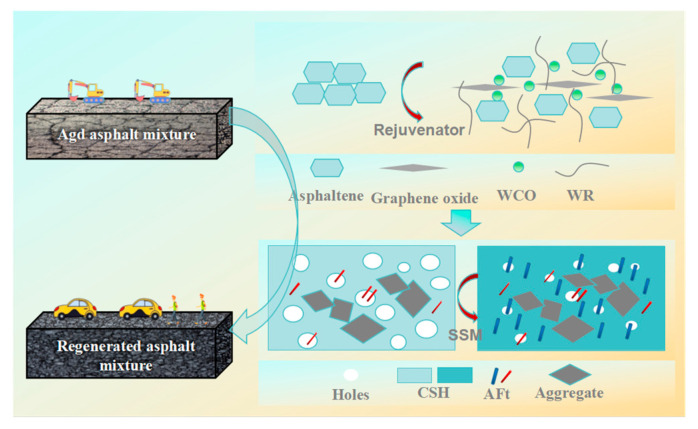
Regeneration mechanism on green and high performance of aging asphalt mixture.

The present study offers a strategy for the environmentally conscious and high-performance regeneration of aged asphalt mixtures, as illustrated in Figure 8. A waste-based asphalt rejuvenator has been developed, comprising primarily waste cooking oil, waste rubber powder, and modified graphene oxide. The incorporation of waste cooking oil can supplement the lightweight components, thereby improving the low-temperature performance of aging asphalt mixtures. The incorporation of waste rubber powder in the rejuvenator has been observed to enhance both the high-temperature and low-temperature performance of aging asphalt mixtures. This can be attributed to the excellent elastoplasticity and low-temperature deformation ability of waste rubber powder [31,32,33]. The addition of modified graphene oxide can facilitate the interlayer flowability of asphalt. The adhesion effect between asphalt and aggregate was thus improved, thereby improving their water stability [34,35]. Solid waste-based solidification materials have been developed to serve as a replacement for cement in the enhancement of asphalt mixture performance. The composite activator within the solidification material can promote the solid waste substrates to generate greater quantities of hydrated calcium silicate gel and needle bar ettringite than would be produced through cement hydration [25]. This results in a more effective cementation effect and improved pore-filling function, which in turn enhances the structural compactness and consequently improves the overall performance of the regenerated asphalt mixture.

## 4. Conclusions

In this study, an aging asphalt mixture was regenerated by the waste-based rejuvenator and cemented by SSMs. A splitting test, wheel tracking test, and three-point bending test were conducted, and the regeneration mechanism was analyzed. Based on the test results, the following conclusions can be drawn:

(1) The properties of the asphalt mixture could be moderately enhanced by the appropriate substitution (30%) of RAP. The splitting strength, dynamic stability and splitting strength ratio were decreased by 13%, 15%, and 5%, respectively, with the 100% substitution of RAP. In this instance, the refinement of recycled aggregate has an adverse effect on the interlocking structure. Furthermore, the aged asphalt coating applied to the surface of the recycled aggregate also has an adverse impact;

(2) With the replacement of cement by SSMs reaching 100%, the compressive strength, dynamic stability, flexural strain, and splitting strength ratio of RAM were increased by 40%, 32%%, 14%, and 8%, respectively. A SEM test revealed that the microstructure of SSM hydration is characterized by the generation of a higher quantity of hydrates than that observed in cement. The main hydrates produced include those of the calcium silicate and ettringite. Furthermore, there is a notable increase in the relative content of ettringite, which contributes to the overall densification of the structural system;

(3) The regeneration mechanism on green and high performance of aging asphalt mixture proposed in this study can be explained in that for the rejuvenator, the waste cooking oil can supplement the lightweight components. The waste rubber powder showed excellent elastoplasticity and low-temperature deformation ability. The modified graphene oxide can facilitate interlayer flowability of asphalt. For SSMs, the composite activator can promote more generation of hydrated calcium silicate and ettringite in solid waste steel slag and fly ash systems. The stronger structural compactness improves the overall performance of the RAM. Future research will evaluate the durability and economic feasibility of RAM. A study on the RAM test section will be conducted. The objective is to verify the performance of the rejuvenator and SSMs in large-scale construction processes and compare it with the results obtained in laboratory tests. This information will be used to optimize the composition of the rejuvenator and SSMs.

## Figures and Tables

**Table 1:** Basic properties of SSM.

Water Content [%]	Fineness/ >80 μm	Initial Setting Time/min	Fluidity/mm			Mortar Strength/MPa	
			Initial	30 min	60 min	7 day	28 day
≤1	≤10	≥45	≥100	≥90	≥80	/	/
0.12	1.5	80	180	160	145	26.3	34.2

**Table 2:** Gradation of emulsified cold-recycled asphalt mixture (30% replacement of RAP).

Cumulative Passing [%]	19	16	13.2	9.5	4.75	2.36	1.18	0.6	0.3	0.05	0.075
AC-16 upper limit	100	100	92	80	62	48	36	26	18	14	8
AC-16 lower limit	100	90	76	60	34	20	13	9	7	5	4
Gradation	100	94	83	75	56	43	32	23	15	11	6

## Data Availability

All data, models, and code generated or used during the study appear in the submitted article.

## Abbreviations

RAP	reclaimed asphalt pavement
SSM	solid waste-based solidification material
RAM	regenerated asphalt mixture by the rejuvenator and SSMs
WCO	waste cooking oil
WR	waste rubber powder
HMA	hot mixed asphalt mixture
CSH	calcium silicate hydrates
Aft	ettringite
AC	asphalt concrete
